# Exploring the Correlation Between Papillary Thyroid Carcinoma (PTC) and the Usage of Oral Contraceptive Pills Among Affected Females

**DOI:** 10.7759/cureus.65998

**Published:** 2024-08-02

**Authors:** Hussain I Alsinni, Bashair Alwasiyah, Ashwag Alwagdani, Mohammed Namenkani, Raghad Althomali, Ahmed S Alsulami, Areej Alsehly, Sultana Khan

**Affiliations:** 1 Otolaryngology - Head and Neck Surgery, Al-Jabr Eye and ENT Hospital, Al-Ahsa, SAU; 2 Otolaryngology - Head and Neck Surgery, King Fahad General Hospital, Jeddah, SAU; 3 Otolaryngology, King Fahad Armed Forces Hospital, Jeddah, SAU; 4 Faculty of Medicine, University of Jeddah, Jeddah, SAU; 5 Faculty of Medicine, King Abdulaziz University, Jeddah, SAU; 6 Graduate Medical Education, Faculty of Medicine, King Abdulaziz University, Jeddah, SAU; 7 Otolaryngology - Head and Neck Surgery, Faculty of Medicine, King Abdulaziz University, Jeddah, SAU; 8 Faculty of Medicine, Ibn Sina National College for Medical Studies, Jeddah, SAU

**Keywords:** thyroid disorders, tertiary hospital, saudi arabia, reproductive factors, public health, hormonal factors, female health, epidemiology, contraceptive use, cancer risk factors

## Abstract

Background: The increasing incidence of papillary thyroid carcinoma (PTC), particularly among women, has prompted an investigation into possible associated factors. The effect of oral contraceptive pill (OCP) usage is debatable, with varying and often conflicting results. It is not confirmed whether OCPs have a protective effect against thyroid cancer or an increased risk.

Objective: The objective of this study is to investigate the prevalence of OCP usage among females diagnosed with PTC at a tertiary hospital in Saudi Arabia.

Methods: The study included females aged 18 and above diagnosed with PTC. An OCP user was defined as a female exposed to OCPs for at least one month. Data collection involved chart reviews and phone interviews, and statistical analyses were conducted using Excel and SPSS.

Results: Among 58 female patients diagnosed with PTC, 29.3% (n=17) reported using OCPs, and 70.7% (n=41) were non-users. The ages of OCP users ranged from 26 to 56 years, with a median age of 44 years. The duration of OCP usage varied from 1 to 72 months, with a median duration of seven months. Additionally, for the non-users of OCPs, the age range was from 21 to 85 years, with a mean age of 46.4 years. The median ages for the total sample, OCP users, and non-users were 43.5, 44, and 43 years respectively. The timing of OCP usage among users varied from 1 to 35, with a mean timing of 13.

Conclusion: The study found about one-third 29.3% (n=17) of patients diagnosed with PTC reported using OCPs. These results contribute to the ongoing debate within epidemiological studies regarding the association between PTC and various reproductive factors, including OCP use. Further research is needed to clarify this relationship and its implications on public health.

## Introduction

Over the past few decades, the incidence of thyroid carcinomas, particularly papillary thyroid carcinoma (PTC), has seen a notable rise, with rates increasing from 4.56 to 14.42 cases per 100,000 individuals between 1975 and 2013 in the United States. Women are disproportionately affected, with incidence rates 3-5 times higher than in men [1].

This escalation is primarily attributed to advancements in medical practices, such as the widespread use of neck ultrasonography since the 1980s, which has led to the detection of previously small and undetected carcinomas [2]. However, while most of this increase can be attributed to micropapillary lesions, there is compelling evidence from several epidemiological studies showing a simultaneous increase in the incidence of larger carcinomas, suggesting a genuine rise in thyroid cancer rates [2].

Numerous risk factors for thyroid cancer have been identified in previous studies, including genetic predisposition, exposure to environmental pollutants and radiation, hormonal influences, elevated levels of thyroid-stimulating hormone (TSH), a prior history of proliferative thyroid conditions, iodine intake, lifestyle factors, obesity, and dietary habits [1]. However, the relationship between thyroid cancer and reproductive factors, such as oral contraceptive (OC) use, pregnancy, parity, menstrual cycle regularity, hysterectomy, oophorectomy, and menopausal status, remains debated [1]. Female sex hormones have been proposed as a potential factor in the increased incidence of thyroid cancer. However, the impact of long-term use of OCs remains uncertain [2]. A case-control study by Cordina-Duverger et al. in France sought to clarify this relationship by examining the influence of hormonal and reproductive factors on the risk of PTC. Their findings suggested that late menarche and early menopause were associated with increased incidence of thyroid cancer, while exposure to exogenous hormones via oral contraceptive pills (OCPs) or hormonal replacement therapy in postmenopausal women decreased the risk [2].

Additional studies, such as a systematic review and meta-analysis of prospective cohort studies from 2015 and a meta-analysis by Mannathazhathu et al. in 2019, also concluded that OCP use may have a protective effect against thyroid cancer [3,4]. However, contrasting findings were reported in a 2023 retrospective cohort study, which suggested that women who consumed OCs containing cyproterone acetate and ethinylestradiol had a higher risk of thyroid cancer than non-users [5]. Further complexity was added by the findings of a cohort study conducted in 2021, which suggested that an increased risk of thyroid cancer may be associated with a variety of indicators of longer reproductive years [6]. Meanwhile, a 1999 meta-analysis of case-control studies found no indication of increased thyroid cancer risk 10 or more years after discontinuing OC use [7]. Given these diverse and sometimes conflicting findings, it is clear that further research is needed. In this study, we aim to determine the prevalence of previous OCP usage among females diagnosed with PTC. Through our investigation, we aspire to provide valuable insights that contribute to a more nuanced understanding of this critical health issue.

## Materials and methods

Study design and setting

This study was designed as a retrospective observational cohort study, conducted at King Fahad Armed Forces Hospital (KFAFH), Jeddah, within the Department of Otolaryngology - Head and Neck Surgery. The research team collected comprehensive data from female patients diagnosed with PTC from 2013 to 2023.

Participants

The study included females aged 18 years and above, irrespective of their marital status, who underwent surgical treatment and were histopathologically confirmed to be cases of PTC. For the purpose of this study, an OCP user was defined as a female who had been exposed to OCPs for a minimum of one month. Females diagnosed with other types of thyroid cancer such as follicular cancer, medullary cancer, or anaplastic thyroid cancer, and women who used contraceptive methods other than OCPs such as intrauterine devices, skin patches, and contraceptive injections were excluded from the study.

Data collection

Data collection involved a thorough chart review using an online data sheet form. The research team conducted phone interviews with each participant to gather information about their previous use of OCPs. All collected data was securely entered into an Excel spreadsheet. Only the co-investigators and primary investigator had access to ensure the participants' information was securely handled.

Statistical analysis

The study employed Microsoft Excel and IBM SPSS Statistics for Windows, Version 29 (Released 2023; IBM Corp., Armonk, New York, United States) for statistical analysis. Excel was employed for initial data organization and preliminary data analysis. In contrast, SPSS was utilized for the computation of descriptive statistics such as means, medians, minimums, maximums, and interquartile ranges, both for the total sample and specifically for the users and non-users of OCs. Frequencies and percentages were calculated for categorical variables like contraceptive use. For continuous variables like age, duration, and timing of contraceptive use, we calculated the minimum, maximum, mean, median, and interquartile ranges (IQRs).

Ethical considerations

Prior to the commencement of the study, ethical approval was obtained from the Medical Services Department for Armed Forces, Scientific Research Center, Research Ethics Committee. The research was conducted in accordance with all relevant ethical guidelines to ensure the protection of participants' confidentiality and rights.

## Results

Our study focused on a sample comprising 58 female patients, all diagnosed with PTC. In the context of contraceptive usage among the study population, Table 1 displays the data in detail. We found that 29.31% (n=17) of the participants reported using OCPs. In contrast, 8.62% (n=5) preferred other contraceptive methods. However, the majority of the participants, 62.07% (n=36), reported not using any contraceptives. It is worth noting that among the total participants, 70.69% (n=41) were non-users of OCPs, whereas 29.31% (n=17) reported using them.

**Table 1:** Distribution of the usage of contraceptives among the study population ^*^OCP: Oral contraceptive pill

Use of contraceptives	N	%
Yes	17	29.3%
Other methods	5	8.6%
No	36	62.1%
Use of oral contraceptives (OCP)*		
No	41	70.7%
Yes	17	29.3%

Moving on to the age distribution of the participants, as shown in Table 2, we observed a range between 21 and 85 years across the total sample, with a mean age of 46 years. Looking more closely at the users of OCPs, their ages varied from 26 to 56 years with a mean of 45.1 years. For the non-users of OCPs, the age range spanned from 21 to 85 years, with a mean age of 46.4 years. The median ages for the total sample, OCP users, and non-users were 43.5, 44, and 43 years respectively. The first IQR for the total sample, OCP users, and non-users was 36, 40, and 35 years respectively, while the third IQR was 55, 54, and 56 years respectively.

**Table 2:** Distribution of age among the total sample and specifically among users and non-users of oral contraceptive pills (OCPs) IQR: Interquartile range

Statistics	Total sample	Use of oral contraceptive pill (OCP)	
		Yes	No
Minimum	21	26	21
First IQR	36	40	35
Mean	46	45.1	46.4
Median	43.5	44	43
Third IQR	55	54	56
Maximum	85	56	85

Finally, Table 3 provides a statistical analysis of the duration and timing of OCP usage among the users. The duration of OCP usage ranged from 1 to 72 months, with a mean duration of 19 months, a median of 7 months, and first and third IQRs of 6 and 24 months respectively. As for the timing of OCP usage, it varied from 1 to 35, with a mean timing of 13, a median of 12, and first and third IQRs of 4 and 21, respectively.

**Table 3:** Statistical analysis of usage duration and timing of oral contraceptive pills (OCPs) among users IQR: Interquartile range

Statistics	Duration (months)	
	When was the OCP last used?	What is the duration of OCP use?
Minimum	1	1
First IQR	4	6
Mean	13	19
Median	12	7
Third IQR	21	24
Maximum	35	72

## Discussion

Our study aimed to explore the correlation between PTC and the usage of OCPs among affected females. We found that 29.31% (n=17) of the participants reported using OCPs, while the majority, 70.69% (n=41), did not use these contraceptives. Our study's findings resonate with and build upon prior research concerning the link between OCP use and thyroid cancer risk, offering fresh perspectives and indicating avenues for further investigation.

The relationship between OCP use and thyroid cancer risk has been a subject of extensive inquiry, yielding varied conclusions. Our findings are consistent with several studies indicating a protective effect of OCP use against thyroid cancer. For instance, a population-based case-control study in France observed a significantly reduced odds ratio (OR) of 0.69 among women who ever used OCs compared to never users, suggesting a protective association [2]. This study also noted a more pronounced reduction in risk among current OCP users, although no dose-response trend with the duration of use was observed [2]. Additionally, another French study found that OCP use showed a nearly significant protective effect irrespective of the duration of use or the time interval between OCP use and the year of reference [8]. Other types of contraceptives, such as patches or implanted devices, were not related to a higher risk of DTC [8]. Similarly, a dose-response meta-analysis of prospective cohort studies reported a summary relative risk (RR) of 0.84 for the longest versus shortest duration categories of OCP use [4]. Additionally, this study revealed a combined RR of 0.96 for each additional year of OCP use, suggesting that prolonged use might reduce thyroid cancer risk [4]. A cross-sectional study using KoGES HEXA data reported adjusted ORs indicating a significant decrease in thyroid cancer risk with OCP use (OR = 0.85) [1]. This aligns with our findings, where a notable proportion of PTC cases were among non-users of OCPs. The Korean study also highlighted the significance of reproductive factors such as the number of children and hysterectomy status in influencing thyroid cancer risk, suggesting that OCP use may interact with other hormonal and reproductive variables in complex ways [1]. The NIH-AARP Diet and Health Study found that women who reported using OCPs for 10 years or more had about half the risk of thyroid cancer compared to never-users [9]. In fact, several studies have observed inverse or no association of differentiated thyroid carcinoma with prolonged OCP use [10-13]. Furthermore, the Nurses’ Health Study II found that among premenopausal women, long-term OCP use (15 or more years) appeared to decrease the risk of thyroid cancer by 46%, although this result did not reach statistical significance [6]. However, modeling the duration of OCP use as a continuous variable revealed a significant linear trend toward increased risk of thyroid cancer with a longer duration of use. These findings collectively suggest a potential protective effect of OCPs against thyroid cancer [6].

However, the relationship between OCP use and thyroid cancer risk is complex and multifaceted. Some studies present a more nuanced or variable relationship. For example, The California Teachers Study noted that among younger women, OCP use was associated with some elevation in risk of PTC, whereas among older women, estrogen-alone therapy but not combined estrogen-progesterone therapy showed similar trends, suggesting age-specific and therapy-specific variations in risk [14]. Another study focusing on specific OCPs containing cyproterone acetate and ethinylestradiol showed a higher risk of thyroid cancer among users [5]. This study involved a large cohort of 49,325 subjects and found that users of these specific OCPs had a significantly higher hazard ratio for thyroid cancer compared to non-users, particularly in the 30 to 39-year age group [5]. This highlights the potential variability in risk associated with different formulations of OCPs and underscores the need for further investigation into the effects of specific OCP formulations on thyroid cancer risk.

Moreover, a case-control study in Chinese women found no significant influence of OCP use on PTC risk after adjusting for common factors [15]. This discrepancy could stem from differences in population characteristics, study design, or unmeasured confounders, emphasizing the importance of considering ethnic and genetic factors that might modulate the effect of OCP use on thyroid cancer risk [15]. Another study examining hormonal factors and PTC risk in women found no significant association between OCP use and the risk of PTC [16].

Our study, while providing valuable insights into the correlation between PTC and OCP usage among affected females, has several limitations that should be considered. The small sample size of 58 patients limits the generalizability of our findings, and the retrospective design introduces potential recall bias and reliance on incomplete medical records. Additionally, we did not differentiate between various OCP formulations, which may have different impacts on thyroid cancer risk, and our study lacked detailed information on the duration and dosage of OCP use. Confounding factors such as genetic predisposition, lifestyle, and other hormonal and reproductive factors were not comprehensively controlled, potentially skewing the results. The absence of a control group precludes direct comparisons, making it difficult to draw robust conclusions about the relative risk of OCP usage. Furthermore, the cross-sectional nature of our study does not allow for establishing causality. Despite these limitations, our study encompasses a number of noteworthy strengths that back up the validity of our findings. These strengths include a thorough ten-year data collection, well-defined participant criteria, exacting data collection techniques, strong statistical analysis, and adherence to ethical guidelines.

## Conclusions

Our findings indicate that a significant portion 29.31% (n=17) of the patients diagnosed with PTC reported using OCPs. This prevalence suggests a possible association between OCP use and PTC. However, a large portion of the participants 70.7% (n=41) were non-users of OCPs, indicating that further factors contribute to the development of PTC. Given these findings regarding the connection between OCP usage and PTC, we recommend further research to explore this association more deeply. Future studies should consider including larger sample sizes and more diverse patient populations to increase the generalizability of the findings. It would also be beneficial to investigate other potential influencing factors such as genetic predisposition, lifestyle choices, and other forms of hormonal influences, to gain a more comprehensive understanding of PTC's etiology.
